# Functional insights from recombinant production of bacterial proteases in *Saccharomyces cerevisiae*

**DOI:** 10.1186/s12934-025-02732-x

**Published:** 2025-05-22

**Authors:** Tova Lindh, Mattias Collin, Rolf Lood, Magnus Carlquist

**Affiliations:** 1 Biotechnology and Applied Microbiology, Department of Process and Life Science Engineering, Lund University, P.O. Box 124, Lund, SE-221 00 Sweden; 2 Genovis AB, Karl Johans väg 104, SE-244 21 Kävlinge, Sweden; 3 Infection Medicine, Department of Clinical Sciences, Lund University, BMC, B14, Lund, SE-221 84 Sweden

**Keywords:** Protease expression, Yeast expression system, Recombinant enzyme production, Bacterial protease engineering, *Saccharomyces cerevisiae* host, CRISPR/Cas9 integration

## Abstract

**Background:**

Proteases are important enzymes in food and pharmaceutical industries, but challenges persist in their recombinant production due to host cell proteome hydrolysis and fitness loss. The development of recombinant expression systems for directed evolution of proteolytic enzymes, and industrial production are desirable. This study evaluated *Saccharomyces cerevisiae* as expression host for three bacterial proteases: BdpK (from *Bdellovibrio bacteriovorus*), IdeS, and SpeB (both from *Streptococcus pyogenes*), each with distinct peptide substrate scopes.

**Results:**

We developed an experimental pipeline for analysis of protease gene expression levels and fitness effects on yeast cultures. Heterologous genes were fused with green fluorescent protein and their expression and effects on cell viability was monitored at the single-cell level by flow cytometry. IdeS-GFP fusion was produced efficiently with a gaussian distribution within the population and without compromising cell growth or viability. BdpK, on the other hand, displayed lower expression level and a more heterogenous distribution that was less stable over time. Production of SpeB was not feasible. Inserting the *speB-GFP* fusion gene resulted in complete growth inhibition and a significantly higher frequency of cells with compromised membrane integrity. Plasmid-based expression was compared with integrated-based expression, revealing higher total expression levels and lower degree of population heterogeneity for the latter.

**Conclusions:**

*S. cerevisiae* was found to be an efficient expression host for the bacterial protease IdeS. In contrast, the expression of BdpK and SpeB faced significant challenges, including lack of activity for BdpK, or imposing a substantial fitness burden on the cells for SpeB, likely due to its broad substrate scope resulting in native protein degradation. The findings of this study provide valuable insights into the limitations and possibilities of yeast as an expression host for bacterial protease production and for studying their physiological effects using yeast as a model eukaryote.

**Supplementary Information:**

The online version contains supplementary material available at 10.1186/s12934-025-02732-x.

## Background

Proteases catalyse proteolysis and are a large family of enzymes [1]. They can be grouped according to catalytic residue, like serine protease and cysteine protease, or by the type of reaction that is catalysed, like endopeptidase (hydrolysing internal peptide bonds), exopeptidase (hydrolysing terminal peptide bonds), and aminopeptidase (hydrolysing amino-terminal peptide bonds) [2, 3]. Industrial applications encompass a wide range of sectors, including the food industry (utilized in dairy processing, baking, and meat tenderization), the detergent industry, the leather industry, and the pharmaceutical industry [4, 5]. The global market of proteases represents 60% of the total enzyme market or between 1.5 and 1.8 billion USD per year [1, 5]. Commercially available enzymes are typically produced using recombinant bacteria (*Escherichia coli*, *Bacillus* spp.) and fungi (*Aspergillus* spp.), achieving enzyme concentrations of up to 30 g/L in fermenters [1, 4, 6]. However, the recombinant expression of proteases in a non-native host poses challenges, as the proteolytic activity can result in the breakdown of the proteome, leading to cytotoxicity and an inability to proliferate [7, 8]. Other challenges associated with recombinant protease expression include formation of inclusion bodies, and aggregation of unfolded proteins. Therefore, the optimal microbial host differ depending on the specific protease to be produced.

Baker’s yeast *Saccharomyces cerevisiae* is a preferred host for numerous recombinant proteins, but its application in protease production has been relatively limited. Nevertheless, yeast presents several advantages that make it a promising candidate as a production host. It holds GRAS (generally regarded as safe) status, and industry-wide knowledge of its physiological behaviour at a large scale is well-established [9–12]. Furthermore, several genetic engineering tools and plasmid-based systems, including the CRISPR/Cas9 system, have been developed [13, 14]. In the case of protease production, previous reports demonstrate the production of up to 40 mg/L of yeast carboxypeptidase (CYP) when the gene PRC1 is placed under the control of the strongly regulated *GAL1*p promoter on a multi-copy plasmid [6, 15].

Here, we assess *S. cerevisiae* CEN.PK as a platform host for the production of the IgG-degrading proteases BdpK, IdeS, and SpeB (Table 1), all used as commercial diagnostic tools for the development of antibodies within the pharmaceutical industry, derived from the predatory bacterium *Bdellovibrio bacteriovorus* [16, 17] and the pathogenic bacterium *Streptococcus pyogenes* [18, 19]. BdpK is a calcium-dependent broad substrate spectrum protease, that cleaves human IgG1 above the hinge region, producing intact Fab and Fc fragments even with mutated hinge regions [20]. SpeB is a broad-spectrum protease and digests IgG in the hinge region, generating the same fragments [18]. IdeS specifically targets human IgG, binding to the Fc region before cleavage [21, 22]. The specific activity of IdeS makes it suitable as a biological pharmaceutical and is approved as a drug against antibody-mediated transplant rejection and several clinical trials are ongoing where IdeS is used against other autoimmune conditions [23–25]. The natively producing bacteria, i.e. *B. bacteriovorus* and *S. pyogenes*, are not suitable for production in the pharmaceutical industry (where *S. pyogenes* is a pathogen), and instead *E. coli* is currently used as recombinant host. However, the production in *E. coli* can suffer from a large fraction of insoluble proteins despite significant optimization efforts with different strains, plasmid systems, induction conditions and lysis methods [26]. *S. cerevisiae* may be a superior production system offering advantages such as high titre and productivity, low fraction of insoluble protein, and it does not produce endotoxins [8, 14]. In addition to improved commercial production, the establishment of yeast as expression host could potentially also be used in the development of improved protease variants with higher selectivity and activity [27–29].

**Table 1:** Bacterial proteases used in the present study. For substrate specificity, the sequence in parenthesis represents the cleavage site in the hinge region of human IgG.

Protease	UniProt Accession number	Origin of protein	Size	Substrate specificity	References
IdeS	F8V4V0	*S. pyogenes*	38 kDa	Human IgG specific, recognises 3D structure and cuts below the hinge region. (CPAPELLG/GPSVF). Not reported to have activity for other proteins.	[21, 22, 25, 30–32]
SpeB	P0C0J0	*S. pyogenes*	28 kDa	Broad substrate specificity, cuts human IgG1 above the hinge region. (KTHT/CPPCPAP)	[18, 19, 25, 26, 32–34]
BdpK	Q6MJY9	*B. bacteriovorus*	32 kDa	Broad substrate specificity, cuts human IgG1 above the hinge region. (KSCDK/THTCPPCP)	[20]

In this study, a flow cytometry method to evaluate expression levels and fitness effects of bacterial proteases was developed and applied to investigate boundaries of *S. cerevisiae* as a production host. Codon-optimized genes for three bacterial proteases were fused with the Green Fluorescent Protein (GFP) gene and subsequently overexpressed under the precise control of the inducible *GAL1*p promoter from multicopy plasmids or chromosomally integrated cassettes using CRISPR/Cas9. Expression levels were monitored at single-cell level and population level with flow cytometry and Western blot. Additionally, to study potential differences in fitness effects, cell membrane integrity and growth kinetics were analysed under induced and non-induced conditions under aerobic batch production.

## Materials and methods

### Strains and media

Plasmids and strains used are listed in Table 2. For sub-cloning of plasmid DNA, *E. coli* strain NEB5α (New England Biolabs, Ipswich, MA, USA) was used. For culturing *E. coli*, Lysogeny Broth (LB) medium containing 10 g/L yeast extract, 5 g/L peptone and 10 g/L NaCl, pH 7.0, supplemented with 100 µg/mL ampicillin (IBI Shelton Scientific, CT, USA) was used. For solid medium, LB was supplemented with 15 g/L agar-agar. Bacterial transformants were selected on LB agar plates containing ampicillin, grown for 16 h at 37 °C. Transformants were cultivated at 37 °C for 16 h in LB supplemented with ampicillin and stored in 25% (v/v) glycerol stocks at -80 °C. Glycerol stocks were recovered by plating on LB agar ampicillin plates before cultivation in liquid LB medium supplemented with ampicillin for 16 h at 37 °C on a rocking table.

**Table 2:** Plasmids and strains used and constructed in this study. Abbreviation MCS = Multi cloning site.

Plasmids	Relevant genotype	Reference
YEplac181	LEU2_2µ (Yeast episomal plasmid)	[35]
pCfB2312	*TEF1*p*-Cas9-CYC1*t*_*kanMX	[36]
pCfB2899	X-2-MarkerFree backbone (Chr X: 194944.195980)	[37]
pCfB3019	gRNA targeting chromosomal site X-2_natMX	[37]
pTL10	pESC-LEU; *GAL1*p*-BdpK-CYC1*t	GenScript
pTL20	pESC-LEU; *GAL1*p*-IdeS-CYC1*t	GenScript
pTL30	pESC-LEU; *GAL1*p*-SpeB-CYC1*t	GenScript
pTL50	pUC57; *yEGFP3*	GenScript
pTL80	pCfB2899; MCS	This study
pTL11	pESC-LEU; *GAL1*p*-BdpK-yEGFP3-CYC1*t	This study
pTL12	pTL80; *GAL1*p*-BdpK-yEGFP3-CYC1*t	This study
pTL21	pESC-LEU; *GAL1*p*-IdeS-yEGFP3-CYC1*t	This study
pTL22	pTL80; *GAL1*p*-IdeS-yEGFP3-CYC1*t	This study
pTL31	pESC-LEU; *GAL1*p*-SpeB-yEGFP3-CYC1*t	This study
pTL32	pTL80; *GAL1*p*-SpeB-yEGFP3-CYC1*t	This study
pTL70	pESC-LEU; *GAL1*p*-yEGFP3-CYC1*t	This study
pTL72	pTL80; *GAL1*p*-yEGFP3-CYC1*t	This study
***S. cerevisiae*** **strain**	**Relevant genotype**	**Reference**
CEN.PK 113-7D	Prototrophic strain	Euroscarf.
CEN.PK 113-16B	ΔLEU2 (auxotrophic strain)	Euroscarf.
TMBTL010	CEN.PK 113-16B; YEplac181	This study
TMBTL016	CEN.PK 113-16B; pTL70	This study
TMBTL017	CEN.PK 113-16B; pTL11	This study
TMBTL018	CEN.PK 113-16B; pTL21	This study
TMBTL019	CEN.PK 113-16B; pTL31	This study
TMBTL030	CEN.PK 113-7D; X-2:: *GAL1*p*-yEGFP3-CYC1*t	This study
TMBTL031	CEN.PK 113-7D; X-2:: *GAL1*p*-BdpK-yEGFP3-CYC1*t	This study
TMBTL032	CEN.PK 113-7D X-2:: *GAL1*p*-IdeS-yEGFP3-CYC1*t	This study
TMBTL033	CEN.PK 113-7D X-2:: *GAL1*p*-SpeB-yEGFP3-CYC1*t	This study

Yeast strains *S. cerevisiae* CEN.PK 113-7D (prototrophic strain) and CEN.PK 113-16B (leucine auxotrophic strain) were recovered from 25% (v/v) glycerol stocks stored at -80 °C by streaking on solid Yeast Peptone Dextrose (YPD) plates containing 10 g/L yeast extract, 20 g/L peptone, 20 g/L glucose, 15 g/L agar-agar, for 2–3 days at 30 °C. 200 µg/mL geneticin (G418; Enzo Biochem, USA) and 100 µg/mL nourseothricin (CloNAT; Jena Bioscience GmbH, Germany) was added to YPD when required. Yeast Nitrogen Base (YNB) medium containing 6.7 g/L Yeast Nitrogen Base without amino acids (Becton, Dickinson and Company, USA), 20 g/L glucose, and 0.1 M potassium buffer (pH 6.5) was used for the yeast cultivations. For solid medium, YNB was supplemented with 15 g/L agar-agar. Yeast Peptone Galactose (YPG) containing 10 g/L yeast extract, 20 g/L peptone, 20 g/L galactose was used when indicated. Yeast transformants were cultivated overnight in YPD or YNB at 30 °C and stored in 25% (v/v) glycerol stocks stored at -80 °C.

All chemicals were purchased from Sigma-Aldrich (or Merck; Missouri, USA) if not otherwise stated and restriction enzymes, ligase and polymerases were purchased from Thermo Fisher Scientific (Waltham, MA, USA).

### Molecular biology and plasmid construction

Standard methods for molecular biology were used according to Sambrook and Russell [38]. For colony PCRs, Dream*Taq* DNA polymerase was used and for cloning PCRs Phusion DNA polymerase was used. All primers used were ordered from Eurofins Genomics (Ebersberg, Germany). GeneJET PCR Purification Kit (Thermo Fisher Scientific, Waltham, MA, USA) was used to purify all PCR products and size analyses of purified PCR fragments was done using agarose gel (0.8%) electrophoresis with MUPID-exU submarine electrophoresis system (Mupid; Tokyo, Japan). All constructed plasmids were verified by Sanger sequencing according to the Eurofins Submission Guide (Eurofins Genomics, Ebersberg, Germany).

The bacterial protease genes for BdpK, IdeS and SpeB were codon optimized for *S. cerevisiae* using the tool JCat (http://www.prodoric.de/JCat) [39] and synthesized at GenScript (Leiden, Netherlands). The synthetic constructs were designed using the software Benchling [Biology Software] (https://benchling.com), where the coding sequences were designed to be under the control of the inducible promoter *GAL1*p and the terminator *CYC1*t placed after the coding sequences. The gene encoding GFP was amplified from plasmid pTL50 using primers with overhangs containing the restriction sited for *Hind*III and *Nhe*I. The PCR product and the plasmid pTL20 were then digested with FastDigest restriction enzymes *Hind*III and *Nhe*I to remove the IdeS gene. The digestion was followed by a ligation using T4 DNA ligase generating the plasmid pTL70. The synthetic construct pTL10, pTL20 and pTL30 containing the protease genes were used to create pTL11, pTL21 and pTL31 containing the proteases fused with GFP. This was done through digestion of the constructed pTL70 with FastDigest *Bam*HI and *Hind*III, and amplification of the protease genes from pTL10, pTL20 and pTL30 with primers containing the restriction sites for *Bam*HI and *Hind*III, followed by a T4 DNA ligase ligation. To build the integration cassettes for chromosomal integration, the backbone of pCfB2899 [37] was amplified with primers containing a multi cloning site (MCS) as overhangs and a self-circulation was done to generate pTL80 with the MCS between the homology sequences matching upstream and downstream of the gRNA recognition site. The whole cassettes containing *GAL1*p-Protease-*yEGFP3-CYC1*t or *GAL1*p*-yEGFP3-CYC1*t were amplified with primers containing restriction sites for *Sac*I and *Pae*I. The PCR products and the plasmid pTL80 were then digested with the same restriction enzymes (*Sac*I and *Pae*I) and four different ligations were done to generate pTL12, pTL22, pTL32 and pTL72. All digestions and ligations followed the manufacturer’s protocols.

### Strain construction

*E. coli* NEB5α was made competent according to the Inoue protocol [40] and used for multiplying all plasmids used. Plasmid DNA was then purified using GeneJET Plasmid Miniprep Kit (Thermo Fisher Scientific, Waltham, MA, US). An altered High-efficiency LiAc protocol [41] was used for the yeast transformations and is described previously [30]. The leucine auxotrophic *S. cerevisiae* strain CEN.PK 113-16B was transformed with pTL11, pTL21, pTL31, pTL70 and YEplac181 in parallel and selected on YNB without amino acids agar plates, generating the strains TMBTL017, TMBTL018, TMBTL019, TMBTL016 and TMBTL010 (Table 2). TMBTL010 acted as a negative control for the plasmid-based strains. For the chromosomal integration of the cassettes containing GFP and proteases from the plasmids pTL12, pTL22, pTL32 and pTL72, the backbone was first digested with the restriction enzyme FastDigest *Not*I to generate linear fragments. The prototrophic *S. cerevisiae* strain CEN.PK 113-7D already containing the Cas9 plasmid (pCfB2312) was transformed with the gRNA plasmid pCfB3019 and the linearized fragments as donor DNA. The same transformation protocol as mentioned above was used and transformants were selected on YPD agar plates supplemented with 200 µg/mL geneticin and 100 µg/mL nourseothricin for the two plasmids. Selected transformants were further verified through colony PCR to assure a correct integration of the donor DNA, or to confirm plasmid presence.

### Shake-flask cultivations and induction

Cultivations of *S. cerevisiae* strains in liquid media were performed at 30 °C and shaking at 180 rpm in an incubator (New Brunswick™ Innova^®^ 43 Incubator Shaker, Eppendorf^®^, Hamburg, Germany). Overnight pre-cultures were inoculated from a single colony in 5 mL YNB medium with 20 g/L glucose in 50 mL conical centrifuge tubes. 250 mL shake flasks were then inoculated to an initial OD_620_ of 0.3 in 25 mL YNB medium. Cell growth was monitored through OD_620_ measurements using a spectrophotometer (Ultrospec 2100 pro UV/Visible spectrophotometer, Amersham Biosciences, Buckinghamshire, United Kingdom) and 620 nm was used for all OD measurements. Samples were taken throughout the cultivations for HPLC, flow cytometry and Western blot and enzyme activity assay. After 24 h of cultivation when the glucose was depleted, the induction of gene expression was performed with a stock solution of 200 g/L galactose added directly to the shake flasks to a final concentration of 20 g/L. This was done to all the strains except for TMBTL019 due to slow growth and the glucose was not depleted after 24 h which was the time of induction. Instead, the cells were harvested in a 50 mL conical centrifuge tube by centrifugation at 3220 g for 5 min and then resuspended in YNB medium with 20 g/L galactose and transferred back to the rinsed shake flask. Sampling continued as before after the induction. Samples not analysed immediately were stored at -20 °C (HPLC) or at -80 °C (Western blot and enzyme activity assay) until used. Biological triplicates were performed on all cultivation experiments.

### Flow cytometry analysis

Flow cytometry analysis of GFP signals and cell permeability through Propidium Iodide (PI) staining was performed using BD Accuri C6 + flow cytometer (BD Biosciences, Franklin Lakes, NJ, USA), as described previously [30]. Cell samples were diluted in 0.5 mL of PBS buffer, pH 7.4, to an OD_620_ between 0.1 and 0.3, stained with PI (10 µg/mL) and incubated 15 min in the dark at room temperature prior to analysis. Fluorescence excitation was made with a blue laser (λ = 488 nm), and the fluorescence emission of GFP and PI were collected at 533/30 nm (FL-1) and 670 LP (FL-3), respectively. The fluidics was set to slow flow rate (14 µl/min), the threshold was set to 80,000 on the forward scatter channel, and 20,000 events were collected. The low speed is used to increase resolution with fewer events per second and to minimize background fluorescence from media and buffer. Mean Fluorescence Intensity (MFI) in the FL1-H channel corresponds to the GFP signal and in the FL3-H channel corresponds to the PI signal and was used to report the mean values of the fluorescence intensities obtained. Data visualization and analysis was made with the FlowJo™ Single Cell Analysis Software v10.8.1 (FlowJo, LLC, v10.8.1, Becton & Dickinson). The autofluorescence level and gates for intact and permeable and GFP + and GFP- cells were defined with the control strain CEN.PK 113-7D and a dead control stained with PI.

### Western blot

For Western blot analyses, the cells were lysed using Y-PER™ Yeast Protein Extraction Reagent (Thermo Fisher Scientific, Cat. No. 78991) according to the manufacturer’s instructions. The frozen cell pellets were thawed and weighed and 2.5 µl Y-PER solution per 1 mg of cell pellet were added and the pellets resuspend. The samples were set to agitate at room temperature for 20 min and cell debris were then collected through centrifugation at 13 000 g for 5 min. The supernatant was transferred to new tubes and used immediately. 10 µl cell lysate was mixed with 10 µl 2x sample buffer supplemented with β-mercaptoethanol (β-ME). For control proteins (FabRICATOR^®^ (IdeS) and FabULOUS™ (SpeB), Genovis AB, Kävlinge, Sweden) 0.1 µg enzyme was mixed with 10 µl 2x sample buffer supplemented with β-ME. All samples were boiled at 95 °C for 5 min and then quickly centrifuged before being loaded on an SDS-PAGE gel (4–15% Tris-Glycine), which was run at 80 mA for 1 h and 10 min. The PVDF membranes were activated with methanol and wet in blotting buffer. The gels were blotted onto the PVDF membranes using a Bio-Rad Transblot Turbo. The membranes were transferred into blocking buffer (5% skim milk powder in PBST (PBS with 0.05% Tween-20)) and incubated for 1 h at room temperature on a shaking table. All incubations and washes were done on a shaking table. The blocking buffer was then discarded, and the membranes incubated with the primary antibody in blocking buffer for 30 min at room temperature. Three different primary antibodies/antisera were used at indicated final concentrations in blocking buffer. Purified polyclonal rabbit anti-GFP 1 µg/mL (Invitrogen), purified monoclonal mouse anti-IdeS 0.25 µg/mL (anti-FabRICATOR^®^, Genovis AB), and custom polyclonal anti-SpeB rabbit antiserum diluted 1:5000. Any unbound primary antibodies were then washed away with 10 mL PBST for 5 min, repeated three times. Next the membranes were incubated 30 min at room temperature in the dark with the secondary antibody in 5 mL blocking buffer. Two different secondary antibodies were used at indicated final concentrations. AlexaFluor 647 Goat anti-rabbit F(ab’)2 IgG at 4 µg/mL (A21246, Life Technologies) was used with anti-GFP and anti-SpeB as primary antibodies. AlexaFluor 680 Donkey anti-goat IgG 4 µg/mL (A21084, Life Technologies) was used against anti-IdeS. Any unbound secondary antibodies were then washed away with 10 mL PBST for 5 min, repeated three times. The membranes were then visualized using a ChemiDoc MP imaging system (Bio-Rad).

### Enzyme assays

Previous results showed that lysis through Y-PER Yeast Protein Extraction Reagent inhibited the protease activity assay, hence the cells were lysed in 50 mM Tris-HCl, pH 7.5 using a bead beater (Precellys24 and Cryolys, Bertin Technologies, Montigny-le-Bretonneux, France) precooled with dry ice as described previously [30]. The frozen pellets were thawed and weighed and 100 µl 50 mM Tris-HCl, pH 7.5 were added per 10 mg cell pellet. Cell pellets were resuspended, and the mixture was transferred into Precellys Lysing kit tubes (VK01, Bertin Technologies, Montigny-le-Bretonneux, France). The bead beater was run at the following settings: 5000 rpm, 3 cycles á 45 s, 30 s paus between cycles and air flow at setting 2 to keep the temperature low. The tubes were then centrifuged at 18 000 g for 1 min and the supernatant was transferred into fresh microcentrifuge tubes, placed on ice, and then used immediately. Monoclonal human IgG (i.e., Trastuzumab, 10 µg), was mixed with 9 µl cell lysate in a microcentrifuge tube and incubated overnight at 37 °C. CaCl_2_ (10 mM, as shown previously by [20]), was added to the samples with BdpK as its activity is calcium dependent and 5 mM DTT was added to the samples with SpeB to create a reduced environment required for activity. Purified proteins produced in *E. coli* were used as positive controls (FabDELLO™, FabRICATOR and FabULOUS). The samples were then diluted with 80 µl TBS and 9 µl was then mixed with 9 µl 2x sample buffer supplemented with β-ME. The dilution was done to obtain 1 µg IgG on the gel. The samples were boiled at 95 °C for 5 min and loaded on an SDS-PAGE gel, (7.5% Mini-PROTEAN^®^ TGX Stain-Free™ Protein Gels, #4568025, Bio-Rad Laboratories, USA). The gel was run at 150 V for 30–40 min to separate the proteins. The gel was visualized to determine if there was any activity against IgG and active enzymes in the lysate.

### Protease effects on yeast proteome

Yeast strains CEN.PK 113-7D and TMBTL030 (7D-GFP, integrated) were cultivated in 5 mL YPD containing 20 g/L glucose in 50 mL conical centrifuge tubes for 16 h. The tubes were centrifuged at 3220 g for 5 min and the supernatant was discarded. The cell pellets were resuspended in 5 mL YPG containing 20 g/L galactose to start the induction and the cells were grown for 4 h at 30 °C and shaking at 180 rpm in an incubator (New Brunswick™ Innova^®^ 43 Incubator Shaker, Eppendorf^®^, Hamburg, Germany). After 4 h the cells were harvested by centrifugation at 3220 g for 15 min and the supernatant was discarded. The cell pellets were kept on ice, resuspended in 50 mM Tris-HCl, pH 7,5 and the cells were then lysed by sonication using the following settings, 5 cycles of 45 s at 75% intensity, 1 min pause between cycles (Bandelin Sonopuls, Buch & Holm, Herlev, Denmark), and kept on ice during the sonication. The tubes were centrifuged at 3220 g for 15 min at 4 °C and the supernatant was transferred to fresh tubes and kept on ice. Further optimization of the sonication protocol is recommended as not all the cells were lysed with the protocol used. Cell lysate (100 µL) was transferred to a flat bottom 96-well plate and either incubated on its own or mixed with 100 arbitrary units of purified protease BdpK, IdeS or SpeB (FabDELLO, FabRICATOR and FabULOUS). CaCl_2_ (10 mM) was added to the samples with BdpK as its activity is calcium dependent and 5 mM DTT was added to the samples with SpeB to create a reduced environment required for activity. The samples were incubated at 30 °C overnight and samples for SDS-PAGE were taken after 2 h and 18 h. 10 µL cell lysate was mixed with 10 µL 2x sample buffer supplemented with β-ME and boiled at 95 °C for 5 min and then quickly centrifuged before being loaded on an SDS-PAGE gel (4–15% Tris-Glycine, Mini-PROTEAN^®^ TGX Stain-Free™ Protein Gels, Bio-Rad Laboratories, USA). The gel was run at 200 V for 30 min to separate the proteins. The gel was visualized to determine if there was any activity against the yeast proteome. Protein quantification was performed after 18 h with Qubit™ Protein Assay Kit (Thermo Fisher Scientific, Cat. No. Q33211) according to the manufacturer’s instructions.

### HPLC analysis

Glucose, galactose, and metabolites (acetate, ethanol and glycerol), was analysed with a Waters HPLC system (Milford, USA) equipped with Aminex HPX-87 H ion exchange column (7.8 × 300 mm, Bio-Rad Laboratories, USA) operating at 60 °C with 5 mM H2SO4 as mobile phase and a flow rate of 0.6 mL/min and a refractive index detector (Waters model 2414; Milford, MA, USA).

### Data analysis and statistics

OD and flow cytometry data from three biological replicates was used to calculate mean values and standard deviations. ΔOD is defined as the difference between OD_620_ six hours after induction and OD_620_ at the time of induction and was used to compare growth after induction was started between the strains used. The specific growth rate (µ) was determined as the slope of the exponential growth phase, linear part, when the natural logarithm of OD_620_ was plotted against time. Before induction µ was calculated between 2 and 12 h and after induction between 26 and 32 h of the cultivation (2 to 6 h after induction was started). The statistic test used to determine significance between specific growth rates and CV-values was Welch’s t-test with *p* < 0.05.

## Results

### Construction of yeast strains over-expressing bacterial proteases

The yeast expression system was designed for intracellular localization to enable flow cytometry analysis of the amount of recombinant protein per cell using a GFP-tag, and for studying the physiological effects of the proteases on the yeast cells. Synthetic genes coding for the proteases covered the entire coding sequences except that the native bacterial signal peptides for secretion were excluded. In the case of SpeB, the protein is naturally produced as an inactive zymogen with a signal peptide for secretion and a pro-domain, which has to be proteolytically processed to become active [42]. While the signal peptide is likely not recognized by yeast, we nevertheless designed the coding DNA sequence to translate into the full amino acid sequence including the pro-domain, approximately 40 kDa in size, corresponding to the zymogen [18]. Keeping the signal peptide and pro-peptide facilitates a correct folding of the protein before it is autocatalytically processed into the around 28 kDa active form of SpeB [25]. As the signal peptide for secretion is not recognized by the yeast, the enzyme stays in the cytosol where it is autocatalytically processed into the active form. SpeB requires reducing conditions to be active [19], which is believed to be in the cytosol of *S. cerevisiae* as the yeast cytosol strongly favours the reduced state of proteins [43].

Protease genes were fused with *yEGFP3 (yeast-enhanced green fluorescent protein)*, which has previously shown to generate a strong signal that can be analysed with flow cytometry [44]. To determine where to place the GFP-tag with minimal impact on protease activity, 3D structures of the proteases were generated with AlphaFold2 through ColabFold [45], and the N- and C-termini were evaluated (data not shown). The GFP-tag was placed at the C-terminus, after taking both the predicted 3D structures and autoproteolytic activity towards the N-terminus into account where the placement of GFP at the C-terminus was believed to not interfere too much with the protease activity.

Protease-GFP fusion genes were cloned in 2µ ori plasmids and transformed into the leucine auxotrophic haploid strain CEN.PK 113-16B, generating strains TMBTL017 (BdpK-GFP, plasmid), TMBTL018 (IdeS-GFP, plasmid), and TMBTL019 (SpeB-GFP, plasmid). A control strain with only GFP was also constructed in parallel, TMBTL016 (GFP, plasmid) (Table 2). The CEN.PK background was chosen since it is recognized to be an excellent host for heterologous protein production, with widespread implementation as background strain in physiological studies [46]. Furthermore, the marker gene (*LEU2*) was chosen to maintain the expression vectors for high-level of heterologous proteins, as shown previously for other proteins [47]. Protease gene expressions were driven with the inducible promoter *GAL1*p to separate the growth and the production phase by controlling glucose and galactose levels in the mineral media. In parallel to the construction of plasmid-bearing strains, a single copy of the expression cassettes were chromosomally integrated in the prototrophic haploid strain CEN.PK113–7D (MATa) using CRISPR/Cas9, as described previously [30], generating strains TMBTL031 (BdpK-GFP, integrated), TMBTL032 (IdeS-GFP, integrated), TMBTL033 (SpeB-GFP, integrated), and the control strain TMBTL030 (GFP, integrated). Similar transformation efficiency was observed for all protease genes as well as the GFP control, except the gene encoding SpeB, which gave a significantly lower number of colonies (data not shown).

### Assessment of growth kinetics under aerobic conditions in minimal medium

**Fig. 1:**
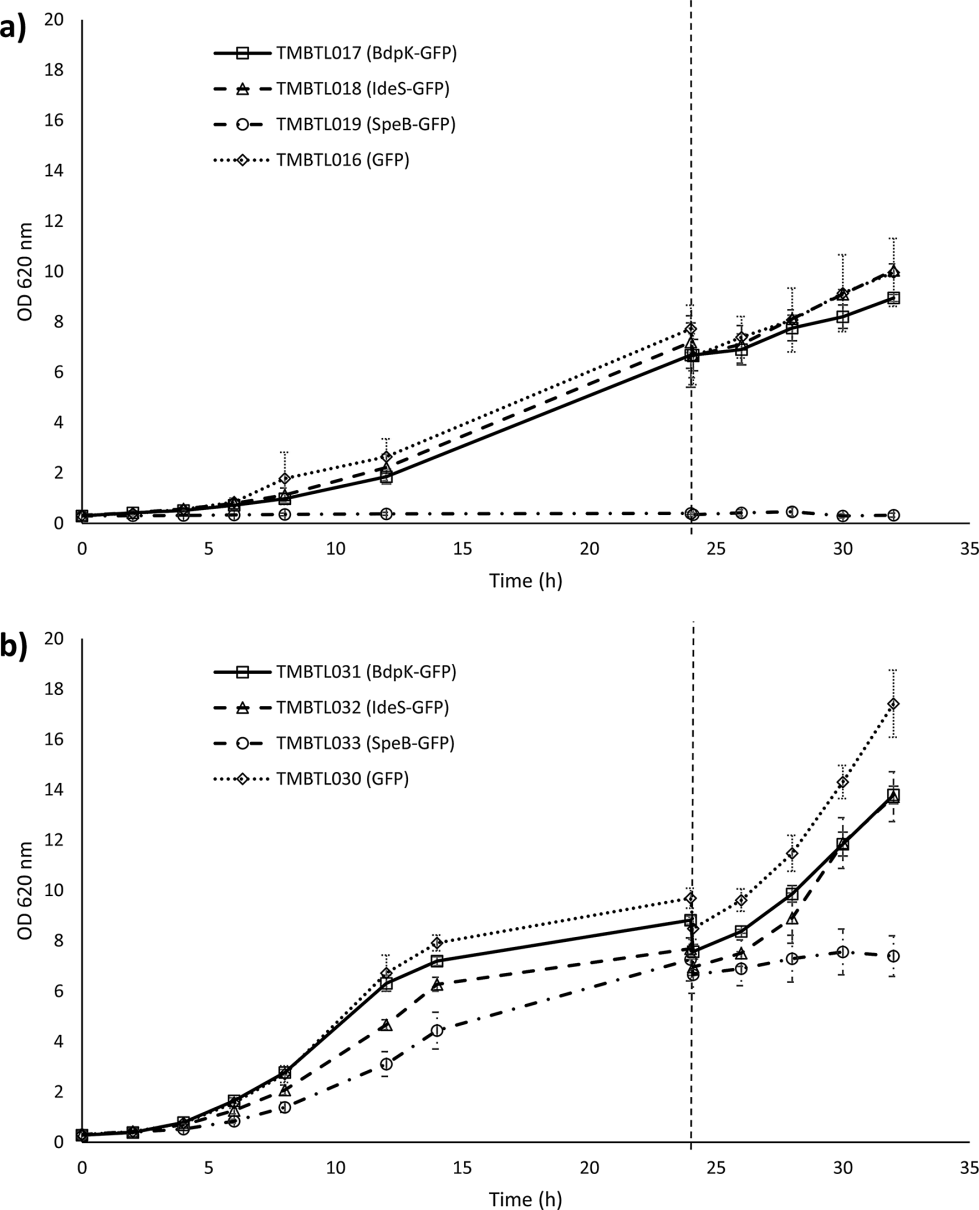
Aerobic batch growth of engineered yeast **a**) plasmid-based, and **b**) integration-based strains in YNB medium before and after induction of the *GAL1* promoter (*GAL1*p) controlling protease gene expression. The dashed vertical line indicates the time of induction where galactose was added to a final concentration of 20 g/L. Symbols represents the strains harbouring the genes for BdpK-GFP (□), IdeS-GFP (∆), SpeB-GFP (○), and GFP control (◊). Error bars correspond to standard deviation of biological triplicates.

Growth kinetics of the constructed strains were evaluated in defined mineral medium without amino acids during aerobic batch cultivation in shake flasks at 30 ºC. Yeast cells were first cultivated with glucose as carbon source to increase the biomass, and upon glucose depletion, the protein production phase was started by the addition of galactose for induction of the *GAL1*p promoter (Fig. 1). Overall, the growth rates were lower during the induction phase, when galactose served as sole carbon and energy source to sustain growth. For the strains TMBTL017 and TMBTL018, which have the proteases BdpK and IdeS fused with GFP on a plasmid, growth rates were similar to the control strain TMBTL016, which only has GFP, both before (µ = 0.15–0.18 h^− 1^) and after induction (µ = 0.043–0.059 h^− 1^) (p-value > 0.05) (Table 3). A significantly lower growth rate, however, was observed for TMBTL019 (SpeB-GFP, plasmid) compared to the control strain (µ = 0.02 h^− 1^ before induction, p-value of 0.014, and µ = 0.0075 h^− 1^ after induction, p-value of 0.038). A similar pattern was observed for the integration-based strains, although the growth rates for these were generally higher than for the plasmid-based strains (Table 3). Strains expressing the protease genes BdpK (TMBTL031) and IdeS (TMBTL032), respectively, had similar specific growth rates as the control strain only expressing GFP (0.23–0.28 h^− 1^ before induction, and 0.084–0.10 h^− 1^ after induction) (Table 3). Of note, in contrast to the corresponding plasmid strain, robust growth occurred for TMBTL033 (SpeB-GFP, integrated) before induction, however at a significantly lower rate compared to the control strain (µ = 0.21 h^− 1^, vs. 0.28 h^− 1^, p-value of 0.036). After induction, the growth of this strain was completely arrested (µ = 0.01 h^− 1^, p-value of 0.0096).

The different impact on yeast fitness by the proteases is also shown in the biomass yield for the production phase, as estimated from OD-values between time of induction and at the end of batch protein production (ΔOD) (Table 3).

**Table 3:** Key parameters for protease production in yeast. Results are obtained from data collected six hours after induction if not otherwise stated. ± corresponds to standard deviation of biological triplicates.

Strain	Heterologous Protein	Plasmid or Integration	Growth rate (1/h)^1^ and *p*-value^2^	ΔOD^3^	MFI total population	MFI GFP+	% GFP+ cells	% intact cells	Coefficient of variation (GFP+ subpopulation)
TMBTL017	BdpK-GFP	Plasmid	0.15 ± 0.005 (0.27) 0.043 ± 0.010 (0.34)	1.5 ± 0.5	5 100 ± 400	40 800 ± 2 000	46 ± 3	98.7 ± 0.54	132 ± 3
TMBTL018	IdeS-GFP	Plasmid	0.16 ± 0.017 (0.40) 0.059 ± 0.016 (0.60)	2.4 ± 0.8	12 000 ± 700	131 000 ± 37 000	55 ± 0.9	94.9 ± 2.7	94 ± 5
TMBTL019	SpeB-GFP	Plasmid	0.02 ± 0.013 (0.014) 0.0075 ± 0.002 (0.0038)	-0.05 ± 0.1	1 900 ± 300	8 600 ± 800	0.16 ± 0.14	65.0 ± 14	-
TMBTL016	GFP	Plasmid	0.18 ± 0.036 0.052 ± 0.006	2.5 ± 0.5	9 400 ± 2 500	94 400 ± 27 000	52 ± 5	94.0 ± 3.8	124 ± 27
TMBTL031	BdpK-GFP	Integration	0.28 ± 0.013 (0.97) 0.084 ± 0.005 (0.21)	4.3 ± 0.6	8 600 ± 900	17 700± 1900	69 ± 11	99.8 ± 0.047	71 ± 1
TMBTL032	IdeS-GFP	Integration	0.23 ± 0.007 (0.061) 0.10 ± 0.002 (0.98)	4.9 ± 0.7	47 700 ± 4 000	50 200± 4 500	99 ± 0.082	99.6 ± 0.12	65 ± 3
TMBTL033	SpeB-GFP	Integration	0.21 ± 0.024 (0.036) 0.01 ± 0.001 (0.0096)	0.9 ± 0.2	600 ± 100	7 200± 2 100	0.086 ± 0.065	98.0 ± 0.76	-
TMBTL030	GFP	Integration	0.28 ± 0.018 0.10 ± 0.013	5.8 ± 0.3	17 200 ± 2 700	24 200± 1 800	87 ± 8.1	99.8 ± 0.047	66 ± 1
TMBTL010	-	Plasmid	0.25 ± 0.014 0.070 ± 0.004	2.9 ± 0.3	500 ± 10	-	-	99.0 ± 0.40	-
CEN.PK 113-7D	-	-	0.29 ± 0.013 0.10 ± 0.007	5.3 ± 0.6	400 ± 6	-	-	99.8 ± 0.047	-

^1^ growth rates were determined for glucose as sole carbon source and after induction for galactose as sole carbon source. Standard deviations are calculated from biological triplicates.

^2^ p-values, presented in parenthesis, were calculated from a Welch’s t-test comparing strain, and corresponding carbon source, with respective control, e.g. TMBTL017, 018, 019 were compared with TMBTL016, and TMBTL031, 032, 033 with TMBTL030 during growth on glucose, or galactose. A p-value < 0.05 was considered to be significant.

^3^ ΔOD is defined as the difference of OD620 six hours after induction and OD620 at the time of induction.

### Single cell analysis of GFP signals through flow cytometry

**Fig. 2:**
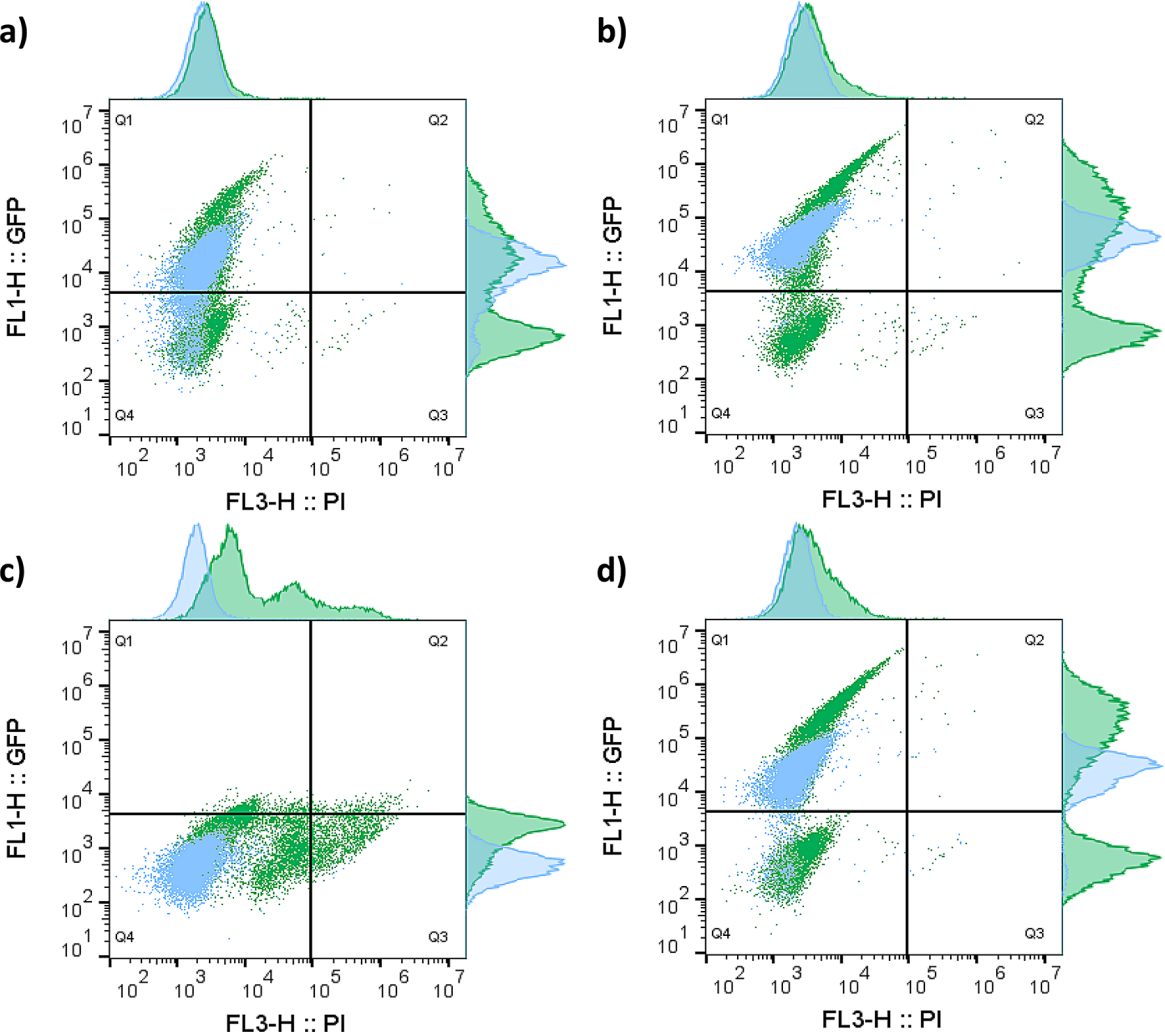
Flow cytometry analysis of yeast strains carrying genes for different proteases fused with GFP. Each dot represents a cell, and the distribution is plotted along with the histograms, showing the fluorescent intensity in the channels FL1-H (GFP) and FL3-H (PI). Q1: intact GFP + cells. Q2: permeable GFP + cells. Q3: permeable GFP- cells. Q4: intact GFP- cells. The green colour corresponds to the multi-copy plasmid-based strains and the blue colour corresponds to the single-copy integrated strains. Samples taken 6 h after induction are representative for three biological triplicates. **a**) BdpK-GFP, strain TMBTL017 (green) and TMBTL031 (blue). **b**) IdeS-GFP, strain TMBTL018 (green) and TMBTL032 (blue). **c**) SpeB-GFP, strain TMBTL019 (green) and TMBTL033 (blue). **d**) GFP control strains TMBTL016 (green) and TMBTL030 (blue).

Flow cytometry analyses were conducted to compare the expression profiles of the GFP-tagged proteases and GFP controls. Yeast cells were stained with propidium iodide (PI), a fluorescent dye that binds to nucleic acids and enters the cell only if the membrane is permeabilized, providing a measure of cell intactness. The combined use of PI and GFP thus constitutes an effective method for assessing the potential adverse effects of the proteases. To assess the dynamics of the expression system, samples were withdrawn regularly and analysed with the FCM experimental pipeline and data processing method, generating time series dynamics capturing the post-induction phase for the different strains (Figure S1). MFI in the FL1-H channel corresponding to GFP signal were highest 8 h after induction for the plasmid-based strains and 4 to 6 h after induction for the single copy integration-based strains (Fig. 2). Bivariate plots were created using the recorded FCM data for all engineered strains, revealing significant differences for the strains, which had either a uniform population with gaussian distribution, or displayed two subpopulations classified as GFP positive (GFP+) and GFP negative (GFP-) (Fig. 2). The coefficient of variation (CV) was calculated for GFP positive subpopulations to quantify the heterogeneity in GFP levels.

**Fig. 3:**
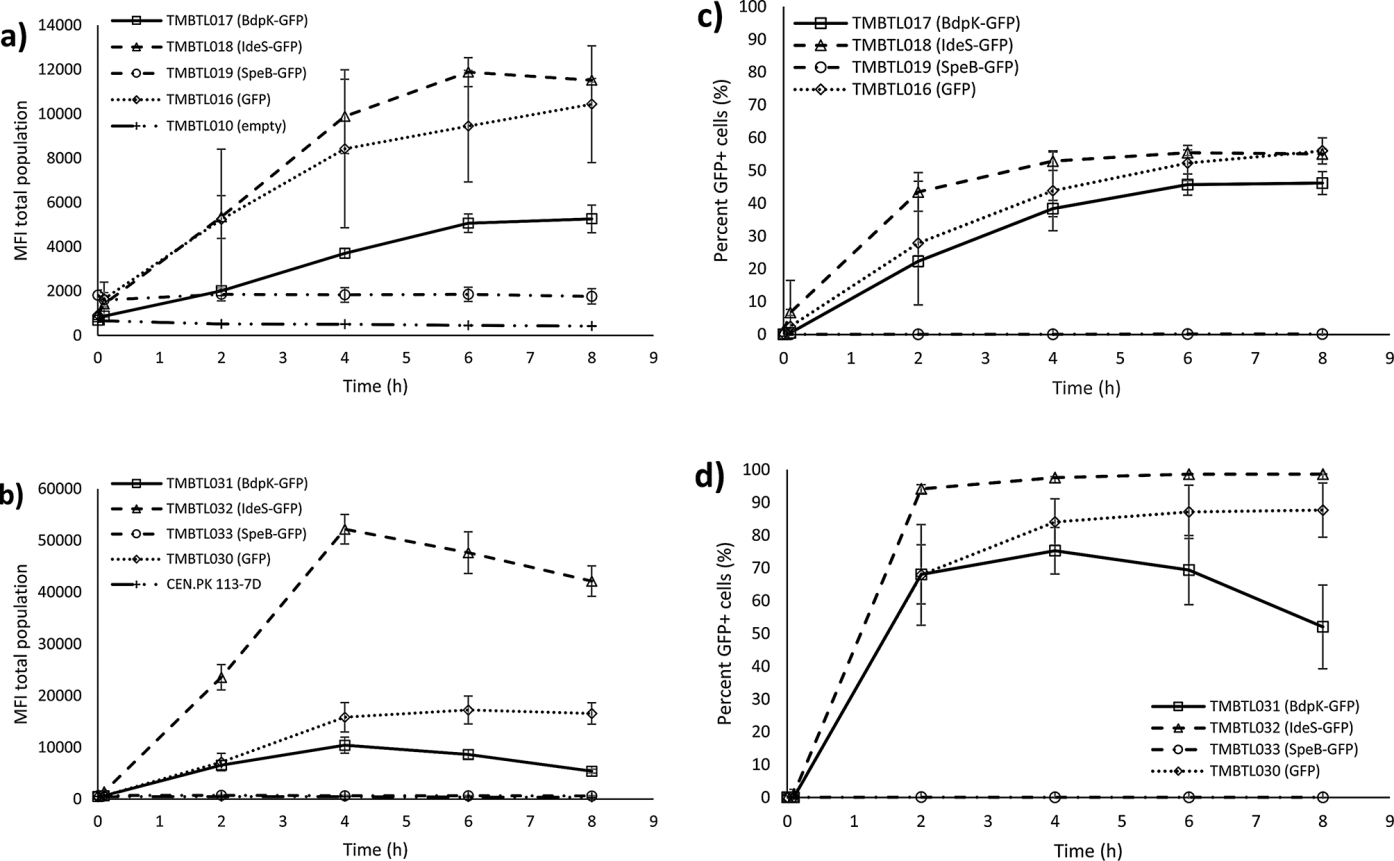
Mean Fluorescence Intensity of FL1-H (GFP) of total yeast populations after induction for **a**) plasmid-based strains, and **b**) integration-based strains. Percentage of GFP + population after induction for **c**) plasmid-based strains, and **d**) integration-based strains. Symbols represent the strains harbouring the genes for BdpK-GFP (□), IdeS-GFP (∆), SpeB-GFP (○), and GFP control (◊). Plus (+) represents the control strains, empty plasmid, or wild type. Error bars correspond to standard deviation of three biological replicates.

Two subpopulations were observed for all plasmid-based strains, except the strain expressing SpeB-GFP. The intactness of the cell membranes was close to 100% for all the strains and can not explain the observed heterogeneity in fluorescence levels, except perhaps for TMBTL019 (SpeB-GFP, plasmid), which displayed around 65% intact cells six hours after induction (Fig. 2 and Figure S3a). TMBTL017 (BdpK-GFP, plasmid) and TMBTL018 (IdeS-GFP, plasmid) produced the highest frequency of GFP + cells (46%, and 55%, respectively) and the highest MFI, which was similar to the control strain TMBTL016 (GFP, plasmid), demonstrating successful production of the GFP-tagged proteins (Fig. 3c). The heterogeneity of GFP levels in the GFP positive subpopulations were similar between strains as observed visually (Fig. 2), and there was no statistically significant difference in CV-values of BdpK-GFP and IdeS-GFP strains compared to the GFP control strain (*p* > 0.05). As mentioned, TMBTL019 (SpeB-GFP, plasmid) did not produce any detectable fluorescence with flow cytometry (less than 0.2% of the cells were classified as GFP + and MFI for this subpopulation was less than 10% of the GFP control) (Table 3). The MFI level of the total population of TMBTL019 (SpeB-GFP, plasmid) was however almost four times higher compared to the autofluorescence level of the negative control strain TMBTL010 (empty plasmid). The higher mean fluorescence can be explained by an increase in cell size, as indicated by an increase in forward scatter (FSC) and side scatter (SSC). The higher autofluorescence level was constant at almost 1900 MFI throughout the experiment and did not increase with the induction (Fig. 3a).

For the integration-based strains, a significantly higher frequency of GFP + cells was observed, with almost 70% for TMBTL031 (BdpK-GFP, integrated), and 99% GFP + cells for TMBTL032 (IdeS-GFP, integrated) (Fig. 3d). MFI levels and CV-values for the GFP positive subpopulations for these strains were in the same range as the control strain TMBTL030 (GFP, integrated). Of note, while the expression levels for IdeS-GFP was stable throughout the induction phase, BdpK-GFP showed a peak level at 4 h, and decreased at a steady rate reaching close to autofluorescence level after 24 h. Similarly to the plasmid-based strain, SpeB-GFP was not produced at detectable levels in TMBTL033 (SpeB, integrated), despite the significant growth loss (Fig. 1b) and reduction in viability (Supplemetary Figure S3b) observed after *GAL1*p induction. MFI, mean FSC, and mean SSC were similar to the values of the GFP negative control strains (TMBTL010 and CEN.PK 113-7D), indicating no difference in cell size for this strain.

The higher MFI of the GFP + population, and the higher degree of heterogeneity (CV-values 94–132 vs. 65–71, *p* < 0.05) in plasmid-based strains compared to the single-copy integration-based strains is likely a gene copy effect (Figure S2). Comparing the two strains expressing BdpK-GFP, the plasmid-based strain (TMBTL017) reached MFI levels 2.3 times of the strain with single-copy gene integration (TMBTL031) (40 800 vs. 17 700 MFI). The strains expressing IdeS-GFP differ in a similar way with GFP + subpopulations for the plasmid-based strain TMBTL018 reaching an MFI 2.6 times of what the integration-based strain TMBTL032 (131 000 vs. 50 200 MFI). However, when the total populations are compared (i.e. both GFP + and GFP- cells), the integration-based strains reached higher MFI levels, which is due to the large amount of non-producing cells for the plasmid-based strains. (Fig. 2).

### Protease production and activity

Western blot using anti-GFP antibody and secondary antibody AlexaFluor647 for signal detection was performed to further investigate the production of the proteins. Yeast cells were harvested 4 h after induction for the integration-based strains and 8 h for plasmid-based strains in accordance with peaking in MFI. Weak signals of the fused protein BdpK-GFP were detected in both the plasmid-based strain TMBTL017 and the integration-based strain TMBTL031, with approximately the same intensity. The fused protein IdeS-GFP was detected in both the plasmid-based strain TMBTL018 and integration-based strain TMBTL032 with a strong signal, where higher protein levels are seen for the plasmid-based strain. The signals of the fused protein BdpK-GFP (lane 2 and 3, strains TMBTL017 and TMBTL031) and IdeS-GFP (lane 4 and 5, strains TMBTL018 and TMBTL032) show partially degraded proteins with varying sizes (Supplementary Figure S4). This was observed in both the plasmid-based strain and the integration-based strain. The fused protein SpeB-GFP gave extremely weak signals in both the plasmid-based strain TMBTL019 and the integration-based strain TMBTL033 and the size of the signal did not correspond to the size of the full fused protein (56 kDa) (Figure S4, lane 6 and 7). The strains TMBTL016 and TMBTL030, which exclusively express GFP, served as controls for the assay and GFP size (27 kDa), revealing higher protein levels in the plasmid-based strain, aligning with the flow cytometry results.

The two-signal blots demonstrated protease production in strains TMBTL018 and TMBTL032 expressing IdeS-GFP, however no signal was obtained for TMBTL019 and TMBTL033 expressing SpeB-GFP (Supplementary Figure S5). The same pattern with a stronger signal for the plasmid-based strain TMBTL018 was observed with the anti-IdeS antibody with signals from the secondary antibody AlexaFluor680 (Figure S5a) and some degraded proteins are visible for both strains as well. The control protein IdeS is 37.7 kDa and adding the size of GFP (27 kDa) the sum corresponds well to the signals of the fused proteins of 65 kDa. With the primary antibody targeting SpeB and the secondary antibody AlexaFluor647 for signal detection, no detectable signals from the strains expressing the fused protein were obtained (Figure S5b). The size of the control protein is 29 kDa and gives a clear visible signal in lane 4 (Figure S6b).

The activities of the fused proteases were evaluated by analysing degradation of human IgG. Crude cell extracts prepared from yeast expressing the bacterial proteases fused with GFP were incubated with human IgG as the substrate 4.5 h and overnight. The results were then visualised on an SDS-PAGE gel (Supplementary Figure S6 (4.5 h incubation) and Figure S7 (overnight incubation)). All the native proteases can cleave human IgG as a substrate [20, 25] and it is important to examine the activity of the recombinantly fused proteases against the same target. All the control proteins showed activity against human IgG in the assay performed, showing that the assay itself worked. However, only one of the fused proteases (IdeS-GFP, lane 6 and 7) showed activity against human IgG. The activity against IgG is visualized by the loss of an intact heavy chain (approximately 50 kDa) and emerging of shorter fragments (approximately 25 kDa) in a distinct pattern. There is no difference between the activity of IdeS-GFP expressed by the plasmid-based strain compared to the integration-based strain. No difference was observed between the samples incubated 4.5 h compared to overnight. No activity was observed in the GFP control strains.

### Bacterial protease effects on yeast proteome

The effects of the bacterial proteases were evaluated by analysing degradation of the yeast proteome through the addition of purified proteases produced in *E. coli* to crude cell extract from the strain CEN.PK 113-7D and TMBTL030 (GFP, integrated). The cell extract mixed with bacterial protease were incubated 2–18 h and the protein degradation was analysed with SDS-PAGE and a Qubit protein quantification assay (Supplementary Figure S8-S10). BdpK and SpeB showed significant degradation of native yeast proteins after 2 h, while no proteolytic activity was observed for IdeS.

## Discussion

Proteases are problematic to produce in non-native hosts due to their proteolytic activity towards the proteome and significant fitness losses of the production host. Here, the potential of *S. cerevisiae* as production host for intracellular production of three bacterial IgG-degrading proteases with narrow and broad substrate scope was evaluated with flow cytometry analysis of the GFP-tagged proteins, and determination of growth fitness effects. Yeast was found to be an efficient host for the production of the IgG-specific protease IdeS, originating from *S. pyogenes*, with minimum effects on cell growth and viability. The fused protein IdeS-GFP was even produced to an even higher extent compared to the GFP control, both for plasmid-based and integration-based strains. Important, IdeS-GFP displayed high activity in cell extracts, demonstrating that yeast indeed is a suitable production host for this protein. BdpK-GFP fusion protein was also possible to produce, although to lesser extent and it was not shown to be active in cell extracts. The broad-spectrum protease SpeB could not be produced, and the introduction of the *speB* gene caused a significant growth defect and decreased viability of the yeast cells.

When comparing plasmid-based to integration-based production, it was found that the latter gave an overall higher production of the proteins considering the entire the population, and the production strains proliferated at a higher rate. Sub-populations characterized by the absence of GFP-fusion expression in plasmid strains were up to 60%, while it was close to zero for the integration-based strains. However, the MFI levels of GFP positive cells were lower for all integration-based strains, possibly attributable to a reduced number of gene copies generally associated with protein expression levels [27]. The expression levels were significantly more widespread in the GFP positive cell of the plasmid strains, which may be explained by a heterogenous number of plasmids per cell. It is well-known that plasmid number varies within a culture and a number between 10 and 40 copies are usually assumed for episomal 2µ based plasmids [48]. For future studies several integrated gene copies could be investigated as an alternative, however it should be noted that a linear correlation between gene copy number and protein yield is not always seen [27].

Loss of plasmids carrying the LEU2 marker gene can be explained by cross-feeding of leucine, or related biosynthetic intermediates, thereby decreasing the selection pressure and allowing for auxotrophic cells to proliferate [47, 49]. Previously, the leucine auxotrophic strain CEN.PK 113-16B, used as the parent strain for the plasmid-based strain, has been shown to generate a mixed culture consisting of both plasmid-bearing and non-bearing cells. To increase the selection pressure and increase the copy number of the plasmids, a deficient promoter e.g., *LEU2*d could be used [47, 48]. The loss of plasmids was particularly noticed during prolonged culturing of TMBTL019, harbouring the plasmid with *speB*, with an almost complete loss of cells carrying plasmids as analysed by comparing number of colony forming units (CFUs) in agar media supplemented with or without leucine (data not shown). SpeB was likely breaking down important parts of the yeast proteome similarly to previous observations for *E. coli*, where most proteins in a lysate solution were degraded [26]. Several proteins in the cell lysate were degraded in the protein degradation assay with pure SpeB added and the effect was seen after 2 h of incubation (Figures S8-S10). The observed fitness impact indicate that important parts of the proteome are targeted by SpeB and as it has been shown to be cytotoxic, the loss of plasmid for this strain might be particularly advantageous for the cells. The slow growth close to zero for TMBTL019 (SpeB-GFP, plasmid) even without induction suggests that trace levels of active enzyme is produced possibly due to promoter leakage before induction. Random activation of the *GAL1*p promoter in individual cells have previously been reported [50] and it seems to be enough to impair the cell fitness. Any promoter leakage before induction is however not measurable with the flow cytometry assay used, probably due to low limit of detection of GFP contra green autofluorescence of the yeast cells as observed previously [51]. A weak signal was obtained in the Western blot assay with the anti-GFP antibody, which indicates that the protein was produced, although the size did not correspond to the full size of the fused protein. It is possible that SpeB has some proteolytic activity against GFP, which would explain why no GFP can be detected with flow cytometry. The protein structure of GFP consists of 4 loops [52], being potential target sites for SpeB, as loops with a loose requirements of amino acid composition are cleaved by SpeB [18]. Previous experiments with octapeptide insertions in these loops have in some cases led to loss of fluorescence [53], suggesting that if cleavage of these loops occurs it could lead to a non-functional GFP. However, this needs to be investigated further. To support the idea that active SpeB was in fact produced, a significant number of cells with compromised membrane integrity was observed in the plasmid-based strain TMBTL019 (carrying SpeB-GFP gene) (ca. 65% of the cells were intact). This was not seen to the same degree for the integration-based strain, TMBTL033 (SpeB-GFP), however, it displayed slower growth rate compared to the negative control before induction and arrest in growth after induction. A way forward to improve the production may be to achieve secretory expression by introducing a leader sequence, which may in addition to reduce cytotoxicity also improve the process by facilitating purification and downstream processing [9, 10]. Another promising strategy may be to compartmentalize the proteolytic activity by localizing the protease in the vacuole. According to the MEROPS peptidase database (http://merops.sanger.ac.uk), *S. cerevisiae* has 122 known or putative proteases [3], and at least seven distinct proteases and their activities have been experimentally established in the vacuole [54]. Some of these proteases have a broad substrate specificity to break down a wide variety of proteins.

The fused protein IdeS-GFP and the strains with only GFP displayed similar patterns of GFP expression after induction. The induction is fast, and the sample taken 2 h after induction with galactose shows a clear increase in MFI and production of GFP. MFI peaks after around 4 h for the integration-based strains and around 8 h for the plasmid-based strains. For protein production purposes, the faster induction of the integration-based strains is advantageous as shorter cultivations are preferable and can decrease production costs [44]. 24 h after induction the cells are still induced, and high levels of MFI are detected. The presumably long half-life of GFP [55] could lead to an accumulation and later stable levels of GFP, leading to higher MFI levels several hours after induction and then plateauing and decreasing in MFI levels. BdpK-GFP peaks in MFI after around 4 h (integrated) and 8 h (plasmid), followed by a descent, suggesting that the fused protein could be degraded (Figure S1). As this is not the case for the strains with only GFP and with IdeS-GFP where only a smaller decrease in MFI is seen after 24 h, it suggests that BdpK could be degrading GFP, which is possible as it is a broad-spectrum protease. If there is an activity against GFP, that could explain the weak signal observed in the Western blot for both strains expressing BdpK-GFP, as it is GFP that is targeted by the antibody used. Degradation of the proteome by BdpK was seen when pure BdpK was added to cell lysate, and similarly to the addition of SpeB, this effect was observed after 2 h of incubation (Figure S7). No drastic fitness burden and decrease in GFP fluorescence were observed for the strains producing BdpK-GFP, indicating that non-vital parts of the proteome are targeted or that an inactive protease is produced. Although the 3D-structure predicted GFP to be positioned without disturbance for the activity of the fused proteins, there is still a chance that the GFP hinders the active site. Another possible explanation of the decrease in fluorescence from BdpK-GFP could be the formation of inclusion bodies and the cells breaking these down, resulting in a lower fluorescence, although, this needs to be further investigated. In the case of SpeB the lack of activity is likely related to the limit of detection of the assay, with very little protease being produced as no GFP is seen, resulting in very low enzyme levels in the cell extract. Another possibility is the formation of inclusion bodies, as mentioned above, or energy utilization resulting in a fitness burden but without active proteases, however, further investigations would be needed to confirm or rule out these scenarios. IdeS is the only protease shown to be active against human IgG in the assay performed and no difference between the plasmid-based and integration-based strain is observed. No signs of proteome degradation were observed when pure IdeS was mixed with cell extract and incubated overnight. With the high specificity of IdeS against the hinge region amino acid sequence CPAPELLG/GPSVF of human IgG, and no proteolytic activity against other proteins, previously described [21, 31], it is expected that no activity against GFP, or any other part of the proteome will take place.

## Conclusion

In conclusion, we have developed an experimental pipeline to evaluate the expression levels and fitness burden of bacterial proteases in *S. cerevisiae*. Using this method, we found that the recombinant expression of the active IgG-degrading protease IdeS was successful in yeast and could be further developed into an efficient production system. However, we were unable to produce the active broad-spectrum proteases BdpK and SpeB. Notably, SpeB had severe negative effects on yeast fitness, completely arresting growth and reducing intact cell count after induction of protein production. Flow cytometry analysis of the BdpK-GFP fusion protein demonstrated successful protein expression, although we could not confirm its activity in an IgG degradation assay. The GFP fusion protein facilitated easy monitoring of protein production and provided insights into population heterogeneity, paving the way for the development of high-throughput screening methods to improve bacterial protease production and study the effects of protease variants on yeast as a model eukaryote.

## Electronic supplementary material

Below is the link to the electronic supplementary material.

Supplementary Material 1

## Data Availability

No datasets were generated or analysed during the current study.
