# Supplementary material for: Functional insights from recombinant production of bacterial proteases in Saccharomyces cerevisiae
Source: Microb Cell Fact. 2025 May 22;24:119. doi: 10.1186/s12934-025-02732-x (PMC12096733; doi:10.1186/s12934-025-02732-x)
Supplement: Supplementary file 1 — Supplementary Material 1 [file 12934_2025_2732_MOESM1_ESM.pdf]

Supplementary information:

Functional Insights from Recombinant Expression of Bacterial Proteases in *Saccharomyces cerevisiae*

Tova Lindh[1,2], Mattias Collin[3], Rolf Lood[2,3], Magnus Carlquist[1]*

[1]Biotechnology and Applied Microbiology, Department of Process and Life Science Engineering, Lund University, P.O. Box 124, SE-221 00 Lund, Sweden

[2]Genovis AB, Kävlinge, Sweden

[3]Infection Medicine, Department of Clinical Sciences, Lund University, BMC, B14, SE-221 84 Lund, Sweden

***Corresponding author:** magnus.carlquist@tmb.lth.se

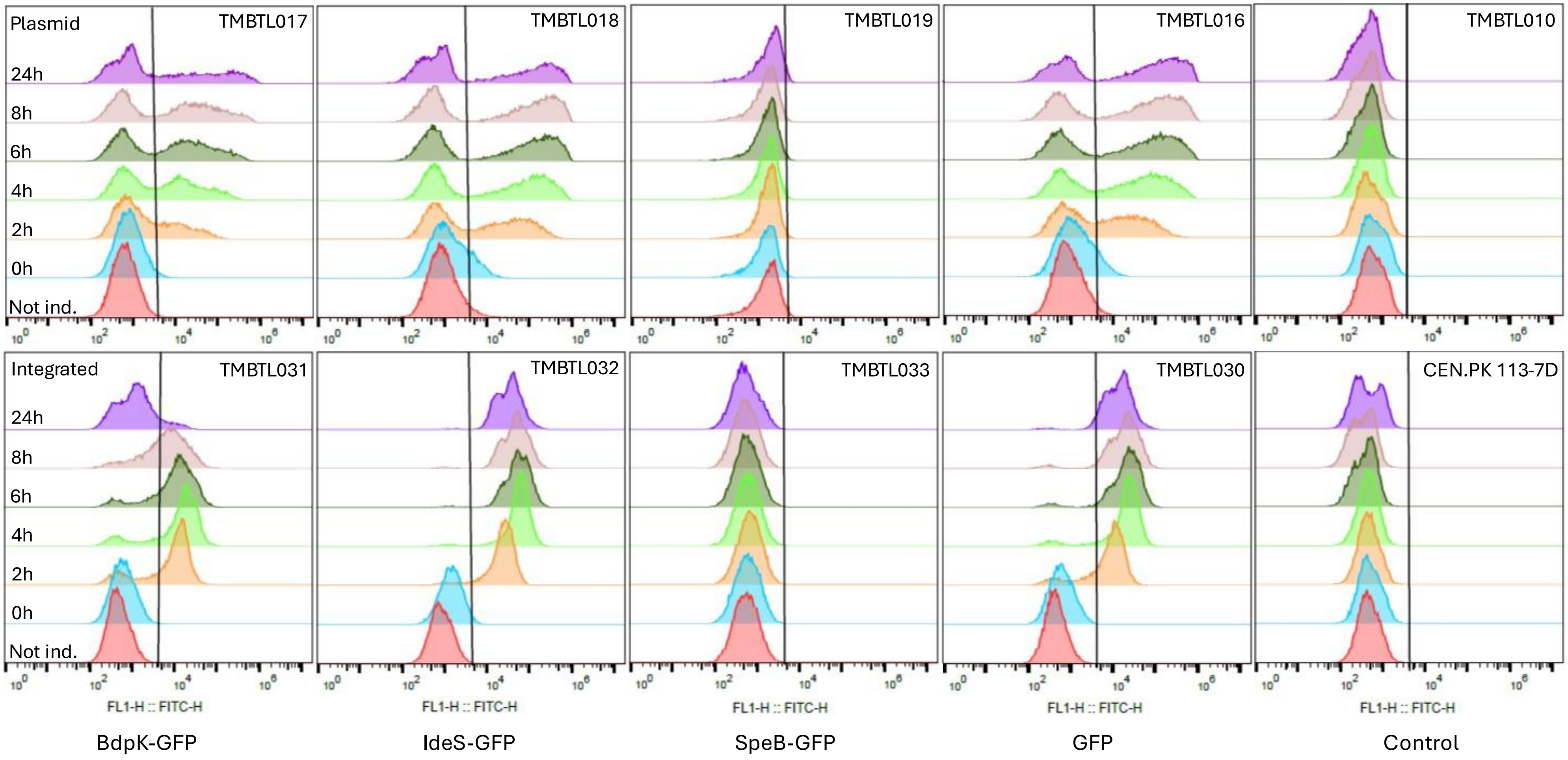

Figure S1: Time series to follow population dynamics after induction for all strain used in the study. The vertical line indicates the threshold set to distinguish GFP- (left) and GFP+ (right) cells. The top row consists of the plasmid-based strains and bottom row consists of the integration-based strains; each strain indicated in the box. Fused protein, GFP and control indicated at the bottom. Each timepoint is indicated on the left. The histograms showing the fluorescent intensity in the channel FL1-H (GFP), revealing population heterogeneity over time after induction with galactose (20 g/L).

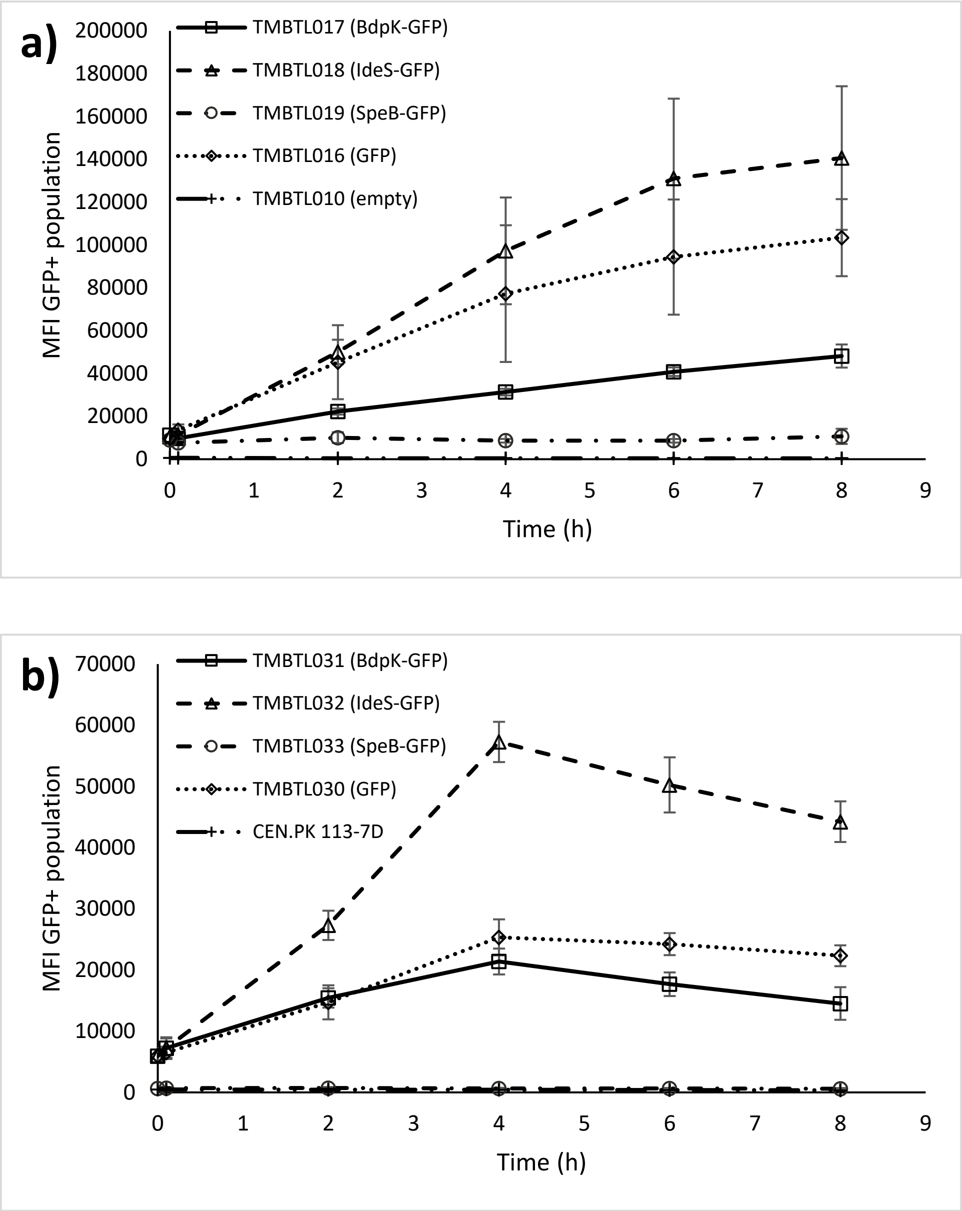

Figure S2: Mean Fluorescence Intensity of FL1-H (GFP) of the GFP positive yeast populations after induction for a) plasmid-based strains, and b) integration-based strains. Symbols represent the strains harbouring the genes for BdpK-GFP (□), IdeS-GFP (Δ), SpeB-GFP (○), and GFP control (◊). Plus (+) represents the control strains, empty plasmid, or wild type. Error bars correspond to standard deviation of three biological replicates.

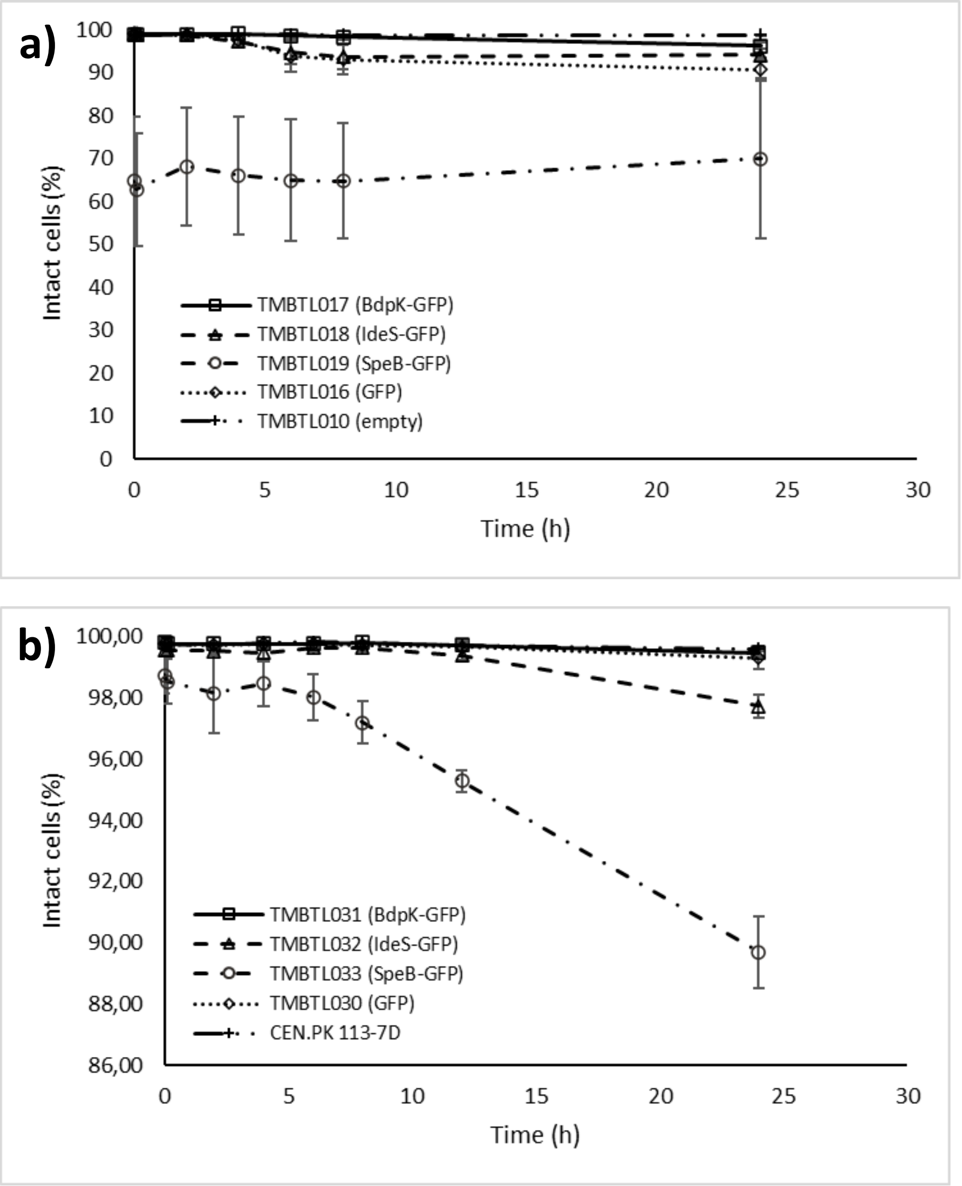

Figure S3: Percent intact cells (%) after induction for a) plasmid-based strains, and b) integration-based strains. Symbols represent the strains harbouring the genes for BdpK-GFP (□), IdeS-GFP (Δ), SpeB-GFP (○), and GFP control (◊). Plus (+) represents the control strains, empty plasmid, or wild type. Error bars correspond to standard deviation of three biological replicates.

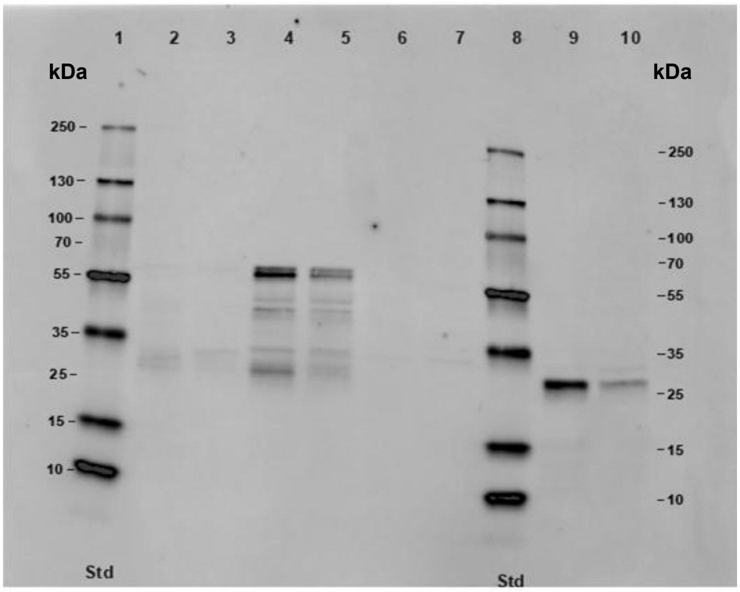

Figure S4: Western blot assay of crude cell extract with an anti-GFP antibody targeting all strains expressing GFP and the secondary antibody conjugated with AlexaFluor647. Cells were harvested 8 hours after induction for plasmid-based strains and 4 hours after induction for the integration-based strains. Lane 1: PageRuler PLUS Prestained (kDa). Lane 2: TMBTL017 (BdpK-GFP, plasmid). Lane 3: TMBTL031 (BdpK-GFP, integrated). Lane 4: TMBTL018 (IdeS-GFP, plasmid). Lane 5: TMBTL032 (IdeS-GFP, integrated). Lane 6: TMBTL019 (SpeB-GFP, plasmid). Lane 7: TMBTL033 (SpeB-GFP, integrated). Lane 8: PageRuler PLUS Prestained (kDa). Lane 9: TMBTL016 (GFP, plasmid). Lane 10: TMBTL030 (GFP, integrated).

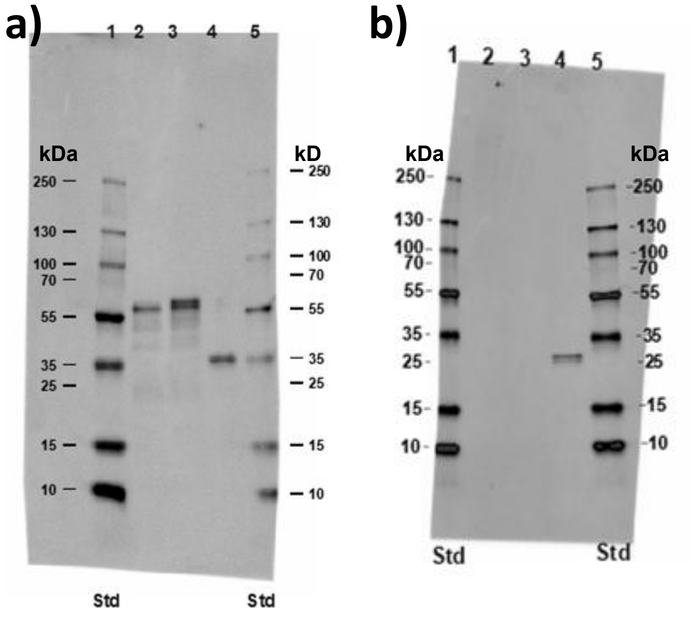

Figure S5: Western blot assay of crude cell extract. Cells were harvested 8 hours after induction for plasmid-based strains and 4 hours after induction for the integration-based strains. Pure enzymes from Genovis AB of FabRICATOR® (IdeS) and FabULOUS™ (SpeB) were used as controls. A) Anti-FabRICATOR® targeting the strains expressing IdeS-GFP and the secondary antibody conjugated with AlexaFluor680. Lane 1: PageRuler PLUS Prestained (kDa). Lane 2: TMBTL018 (IdeS-GFP, plasmid). Lane 3: TMBTL032 (IdeS-GFP, integrated). Lane 4: positive control, pure IdeS. Lane 5: PageRuler PLUS Prestained (kDa). B) Spe-B antiserum targeting the strains expressing SpeB-GFP and the secondary antibody conjugated with AlexaFluor647. Lane 1: PageRuler PLUS Prestained (kDa). Lane 2: TMBTL019 (SpeB-GFP, plasmid). Lane 3: TMBTL033 (SpeB-GFP, integrated). Lane 4: positive control, pure SpeB. Lane 5: PageRuler PLUS Prestained (kDa).

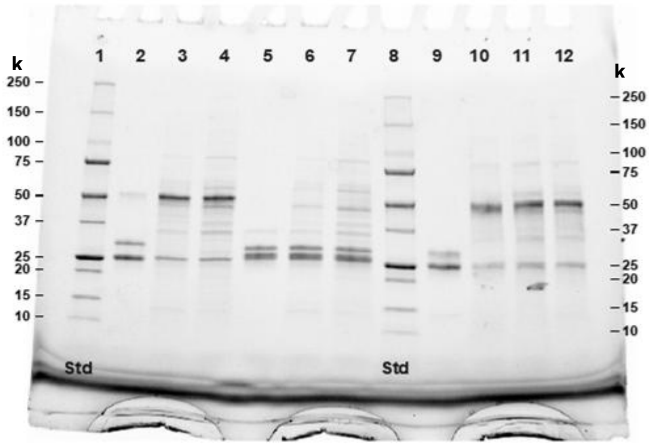

Figure S6: SDS-PAGE analysis of trastuzumab (Herceptin®, human IgG1) for control of digestion by fused proteins in crude cell extract. Samples incubated 4.5 hours. Cells were harvested 8 hours after induction for plasmid-based strains and 4 hours after induction for the integration-based strains. Lane 1: Precision Plus Protein™ Standards (kDa). Lane 2: positive control, pure BdpK and human IgG (digested). Lane 3: TMBTL017 (BdpK-GFP, plasmid) and human IgG (undigested). Lane 4: TMBTL031 (BdpK-GFP, integrated) and human IgG (undigested). Lane 5: positive control, pure IdeS and human IgG (digested). Lane 6: TMBTL018 (IdeS-GFP, plasmid) and human IgG (digested). Lane 7: TMBTL032 (IdeS-GFP, integrated) and human IgG (digested). Lane 8: Precision Plus Protein™ Standards (kDa). Lane 9: positive control, pure SpeB and human IgG (digested). Lane 10: TMBTL019 (SpeB-GFP, plasmid) and human IgG (undigested). Lane 11: TMBTL033 (SpeB-GFP, integrated) and human IgG (undigested). Lane 12: negative control, TMBTL016 (GFP, plasmid) and human IgG (undigested).

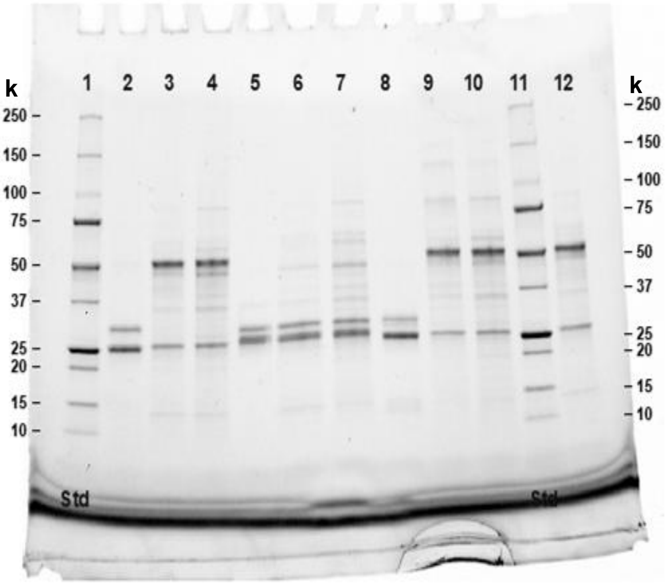

Figure S7: SDS-PAGE analysis of trastuzumab (Herceptin®, human IgG1) for control of digestion by fused proteins in crude cell extract. Samples incubated overnight. Cells were harvested 8 hours after induction for plasmid-based strains and 4 hours after induction for the integration-based strains. Lane 1: Precision Plus Protein™ Standards (kDa). Lane 2: positive control, pure BdpK and human IgG (digested). Lane 3: TMBTL017 (BdpK-GFP, plasmid) and human IgG (undigested). Lane 4: TMBTL031 (BdpK-GFP, integrated) and human IgG (undigested). Lane 5: positive control, pure IdeS and human IgG (digested). Lane 6: TMBTL018 (IdeS-GFP, plasmid) and human IgG (digested). Lane 7: TMBTL032 (IdeS-GFP, integrated) and human IgG (digested). Lane 8: positive control, pure SpeB and human IgG (digested). Lane 9: TMBTL019 (SpeB-GFP, plasmid) and human IgG (undigested). Lane 10: TMBTL033 (SpeB-GFP, integrated) and human IgG (undigested). Lane 11: Precision Plus Protein™ Standards (kDa). Lane 12: negative control, TMBTL030 (GFP, integrated) and human IgG (undigested).

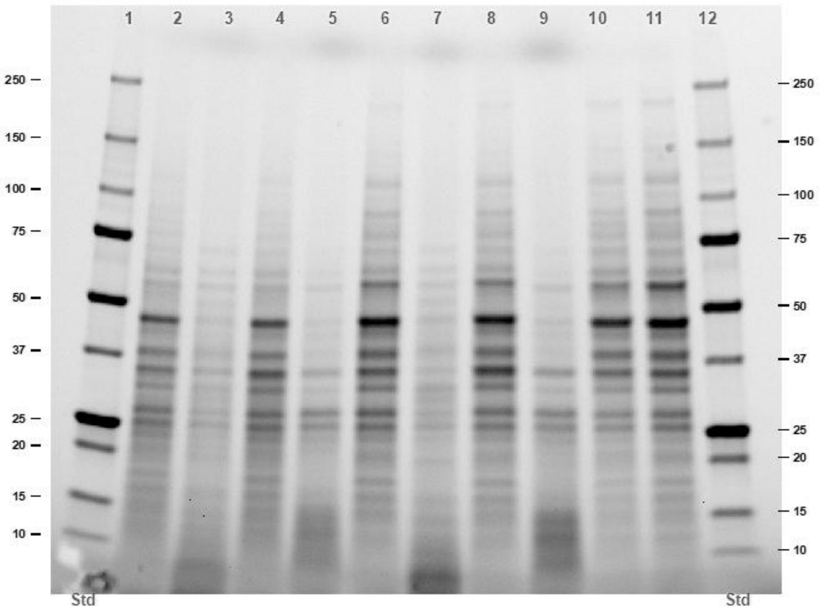

Figure S8: SDS-PAGE analysis of effects on the yeast proteome after 2-hour incubation of crude cell extract with bacterial proteases added. Lane 1: Precision Plus Protein™ Standards (kDa). Lane 2: CEN.PK 113-7D, no protease added (intact proteins). Lane 3: CEN.PK 113-7D with BdpK added (degraded proteins). Lane 4: CEN.PK 113-7D with IdeS added (intact proteins). Lane 5: CEN.PK 113-7D with SpeB added (degraded proteins). Lane 6: TMBTL030, no protease added (intact proteins). Lane 7: TMBTL030 with BdpK added (degraded proteins). Lane 8: TMBTL030 with IdeS added (intact proteins). Lane 9: TMBTL030 with SpeB added (degraded proteins). Lane 10: CEN.PK 113-7D, lysate frozen directly after lysis (intact proteins). Lane 11: TMBTL030, lysate frozen directly after lysis (intact proteins). Lane 12: Precision Plus Protein™ Standards (kDa).

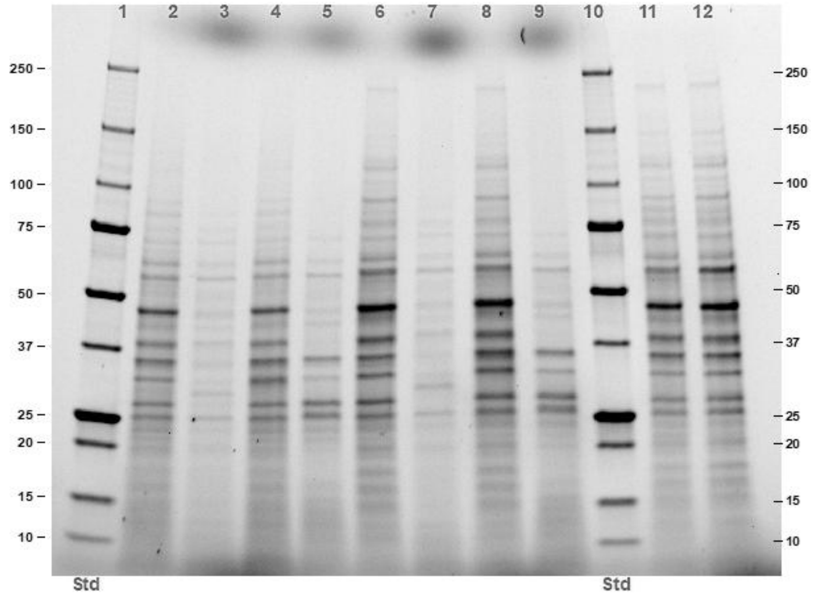

Figure S9: SDS-PAGE analysis of effects on the yeast proteome after 18-hour incubation of crude cell extract with bacterial proteases added. Lane 1: Precision Plus Protein™ Standards (kDa). Lane 2: CEN.PK 113-7D, no protease added (intact proteins). Lane 3: CEN.PK 113-7D with BdpK added (degraded proteins). Lane 4: CEN.PK 113-7D with IdeS added (intact proteins). Lane 5: CEN.PK 113-7D with SpeB added (degraded proteins). Lane 6: TMBTL030, no protease added (intact proteins). Lane 7: TMBTL030 with BdpK added (degraded proteins). Lane 8: TMBTL030 with IdeS added (intact proteins). Lane 9: TMBTL030 with SpeB added (degraded proteins). Lane 10: Precision Plus Protein™ Standards (kDa). Lane 11: CEN.PK 113-7D, lysate frozen directly after lysis (intact proteins). Lane 12: TMBTL030, lysate frozen directly after lysis (intact proteins).

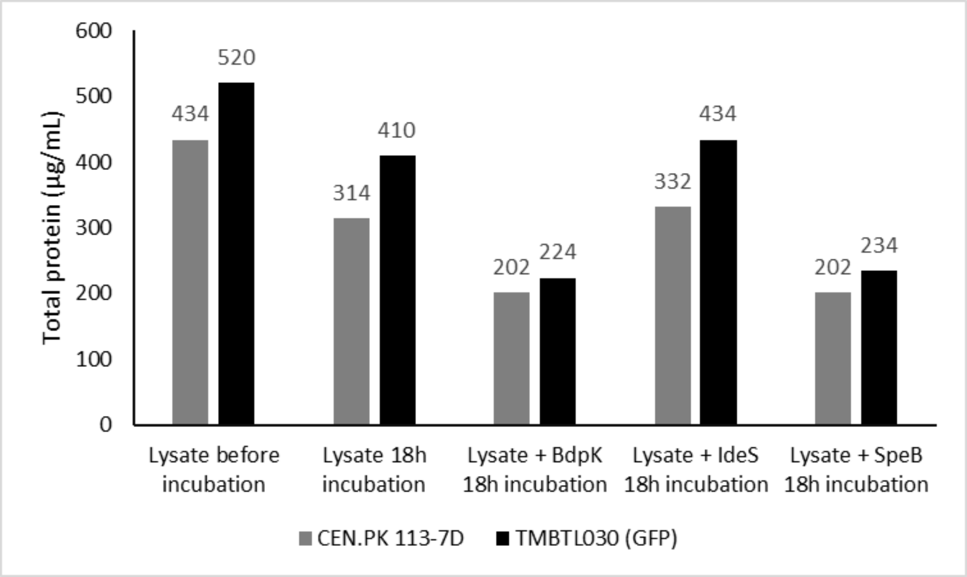

Figure S10: Qubit analysis of effects on the yeast proteome after 18-hour incubation of crude cell extract with bacterial proteases added.
